# The Impact of Big Data Management Capabilities on the Performance of Manufacturing Firms in Asian Economy During COVID-19: The Mediating Role of Organizational Agility and Moderating Role of Information Technology Capability

**DOI:** 10.3389/fpsyg.2022.833026

**Published:** 2022-07-06

**Authors:** Junling Zhang, Hualong Li

**Affiliations:** ^1^Faculty of Economics and Management, East China Normal University, Shanghai, China; ^2^College of Economics and Management, Southwest University, Chongqing, China

**Keywords:** COVID-19, big data, agility, information technology, manufacturing firms, China

## Abstract

The main purpose of this study is to examine the impact of the big data management capabilities on the performance of manufacturing firms in the Asian Economy during coronavirus disease 2019 (COVID-19). In addition to this, this study is also planned to examine the mediating role of organizational agility in the relationship between the big data management capabilities and the performance of Chinese manufacturing firms during COVID-19. Last, this study has examined the moderating role of information technology capability in the relationship between the big data management capabilities and performance of Chinese manufacturing firms during COVID-19. This study adopted the quantitative method of research with a cross-sectional technique. This study employed a questionnaire to gather the data as a research instrument. This study has used the purposive sampling method by keeping in mind the context of this study. Employees of the Chinese SMEs that were at least 10 years old were the population of this study. The research model was being analyzed by employing the “partial least squares” technique through statistical software the Smart PLS version 3. The results are in line with the proposed hypothesis. This study contributed to the literature by suggesting characteristics that promote or prevent the organization from successfully implementing big data and pointed out that showing resistance in information management system implementation may have different effects on the organization. Besides, the study also discussed the relationship between such information systems and the organization. Findings of these two factors provide insights for the practitioners and researchers in assessing the success or failure of organizations for using big data.

## Background

Big data have gained huge popularity due to advancements in hardware and software technologies. Numerous practitioners and scholars have been attracted to big data, as it is expected to be a big thing in the coming era of management. Scholars have also a new term for big data, i.e., next management revolution. Among business communities and scholars, big data analytics (BDA) has become one of the emerging concepts as it has revolutionized statistical tools and is adding both non-financial and financial value to firms. Several white papers and articles have been presented on the potential and current BDA applications. According to a report, businesses have been planning to make investments in business intelligence and analytics tools to enhance HR capabilities in the future. According to the survey, the chief information officers are going to face a huge challenge in meeting digitization aims and this challenge would be the talent gap that is expected to emerge in the coming years. In a survey conducted by, it is revealed that in view of 63% executives, it is essential to hire talent who possesses the required expertise as it is essential to cope with the existing talent gap.

Classical business models and organizations’ knowledge management have significantly evolved with the emergence of big data (Khan and Vorley, 2017; Pauleen and Wang, 2017; Şerban, 2017; Rialti et al., 2019). Big data refers to a heterogeneous and large set of data that involve information in various quantities and types (Lioutas and Charatsari, 2020). Big data allows managers to know well about their competitors, customers, and their own organization. Particularly, using big data enables effective monitoring of internal processes, supply chain processes, and the performance of all processes, assets, and business units within the organization (Rialti et al., 2019). Through big data, updated as well as new data can also be accessed to get information about the competitors’ potential maneuvers (Cappa et al., 2021). Furthermore, it also provides information to the producers concerning the complaints, requests, and behavioral patterns of customers at an individual and aggregate level, i.e., information regarding customer base, and how individual customer behavior changes over time (Liu et al., 2021). Therefore, big data analytic capabilities must be developed by the organizations for selecting relevant information and then processing it to take decisions based on this information. Organizational BDA capabilities refer to a set of capabilities involving personnel capabilities, infrastructure flexibility, and the management capabilities (Korherr and Kanbach, 2021). Promoting BDA capabilities bring about various outcomes. However, several prior studies have reported the effects of BDA capabilities on the economic performance of the organization. Such as, a study discussed the impact of BDA capabilities on the marketing capabilities of an organization, as well as its ability to timely respond and create new strategies. This study also revealed that the performance of an organization can be improved by obtaining new information regarding its customers. On the contrary, a few studies also analyzed how the emergence of BDA capabilities has transformed the supply chain management. Gangwar (2018) also emphasized the role of BDA in improving the organization’s internal processes and operations, such as, its efficiency. In Korherr and Kanbach (2021) study, they analyzed ways through which BDA capabilities influence the organization’s dynamic capabilities. Thus, through its impact on the organization’s adaptability and capability, the big data influences the performance of an organization. The key beneficiary of BDA and big data is the large organizations since they are usually in a good position to easily adopt and process it (Korherr and Kanbach, 2021). Although the literature provides evidence concerning BDA and its effects, still there is an existing gap in the literature. It is only recently that scholars have started realizing the significance of BDA and are trying to develop understanding of the relationships between organizational performance and BDA development. At the same time, research in this area is mostly qualitative and theoretical and is still at the infancy stage in context to qualitative research (Korherr and Kanbach, 2021; Vaio et al., 2021). Therefore, it clearly gives rise to the demand for more research especially the investigation of those areas or traits that are greatly influenced by the organization’s BDA capabilities, and how performance is affected by these capabilities. Moreover, factors that impede BDA implementation must also be identified. This study aims to fill this research gap. The objective of this study is to empirically test the proposed hypotheses, which assume factors that are likely to influence the organizational performance—big data relationship.

This study is conducted to identify the impact of two moderators (i.e., the resistance of organization to implement IMS and the relationship between organization and such systems) and two mediators. Large organizations are the main emphasis of this study, since they are the key beneficiaries and consumers of big data (Mauro et al., 2018). Furthermore, major investments are required to develop BDA capabilities, and such investments can only be made by big firms (Vaio et al., 2021). In an endeavor to cover the identified research gap, undermentioned research questions are designed in the selected area:

– To examine the impact of big data management capabilities (BDMC) on the firm performance during coronavirus disease 2019 (COVID-19).
– To examine the mediating role of organizational agility in the relationship between the BDMC and firm performance during COVID-19.
– To examine the moderating role of information technology capabilities in the relationship between the BDMC and firm performance during COVID-19.

## Hypothesis Development

The resource-based theory posits that it is the valuable, rare, and hard to imitate resources that create competition between the organizations (Barney, 1991). According to Grant (1996), organizational resources are of three types, namely, intangible resources, human resources, and tangible resources, where tangible resources can both be sold or bought as an asset. Intangible resources are the non-physical resources, for instance, knowledge-based resources, while the resources that come under human resources are the relationships, skills, experiences, employees training skills, etc. In organizations, it is the commonly used theory as it explains and predicts the relationships within the organization (Barney et al., 2011). Resource-based theory also suggests that the performance of the firm mainly depends on the organizational resources (Wade and Hulland, 2004). In accordance with the literature on big data and IT capabilities, this study analyzed organizational big data analytic capabilities (BDAC) using specified resources related to big data, such as basic resources, technology, data, managerial skills, data skills, data-driven culture, data quality, domain knowledge, and bigness of data (Wang and Strong, 1996; Gupta and George, 2016). In addition, based on the Grant’s classification, basic resources, data resources, and technology fall under tangible resources, while data-driven culture, the bigness of data, and data quality are classified as intangible resources; and domain knowledge, technical, and managerial skills are classified as the human resources.

Traditional datasets and big data files are different from each other in seven ways, which include veracity, velocity, volume, visualization, variety, variability, and value. Big data refer to the complex and large datasets, for which the traditional statistical models are usually insufficient or cannot provide efficient outcomes (Lioutas and Charatsari, 2020). Organizations face several challenges in managing big data. For information utilization, big data architectures are needed to be designed. Rialti et al. (2019) defined big data architectures as the network of machines, datasets, and processors which collect, store, analyze, and process big data. Big data architectures use data lakes for storing data at every scale and in its original format. The term data lake refers to a repository or system that is used for storing the enterprise’s unstructured or structured data, which can take the form of transformed data from internal processes and monitoring machines or in the form of raw source system data (Rialti and Marzi, 2020). In addition, nested computer networks are also used by the organizations which enable the company to simultaneously process various kinds of data. Nested computer networks are operated on the basis of open-source software, which allows parallel computing and operability between the organizations. Jensen et al. (2019) argue that such features assist in categorizing, collecting, analyzing, and storing data in the enterprise’s repositories.

According to Vaio et al. (2021), data architectures are required to be agile, so they could quickly adapt when changes occur in the organizational structures. Given the big data management complexity, machinery does not seem to be sufficient in this regard and requires professional training or hiring of professionals to perform such tasks (Korherr and Kanbach, 2021). Scientists, engineers, and BDA must be proficient in models which are based on the paradigm of artificial intelligence, such as schema-less data retrieval, Not Only SQL (NoSQL) data models, R, Hadoop, and R (Zhang and Lam, 2019). Thus, in order to deal with challenges arising from big data, simple personnel retraining would be insufficient. Rather, the organization’s entire culture must adapt to the big data culture paradigm (Rialti and Marzi, 2020). According to this paradigm, business-related decisions can solely be relied on the complete or partial utilization of data and machines (Rialti et al., 2019). Hallikainen et al. (2020) argue that computer-aided decision-making should be encouraged among managers to acquire big data benefits.

Besides, another concept, i.e., organizational BDA capabilities was also outlined by Wamba et al. (2017) and defined it as the set of capabilities that enable to deploy and mobilize combination of capabilities and resources, including BDA-based resources. According to Wamba et al. (2017), an organization must develop three fundamental capabilities, these are: (i) BDA personnel capabilities, (ii) BDA management capabilities, and (iii) BDA infrastructure flexibility. There are various reasons why personnel is needed to be skilled; these are: (i) having such skills in people minimizes the chance of BDA rejection and allows integrating new information management system (IMS) for smooth BDA functioning; (ii) since data analysis is usually done by the employees, therefore, they need to be skilled so they could make the right choice for data analysis and then draw conclusions based on their assessments (Korherr and Kanbach, 2021); and (iii) managers are responsible for choosing appropriate technical solution according to the needs of the organization. For this purpose, managers need to learn data analytics skills since such skills help in decision-making, particularly in the case of new data (Kiradoo, 2019).

Big data analytics managerial capabilities play a key role in the selection and implementation of the right information and BDA infrastructure from the dataset. Finally, the BDA infrastructure refers to the information systems which can collect, store, analyze, and process big data and is fundamental for the organization since it ensures smooth data flow and processing under various situations with the help of technologies (Vaio et al., 2021). The literature suggests that developing such capabilities can be helpful for the organization to achieve competitive advantage (Şerban, 2017).

An organization needs to make a huge investment to gain the benefits of BDA. Raguseo and Vitari (2018) argue that several small and medium enterprises (SMEs) do not have enough capital to invest in such systems such as data lakes and parallel computing and do not have the capability to retrain or hire the required personnel for that system. Therefore, only large firms usually gain the advantages arising from BDA. For instance, Ylijoki and Porras (2018) mentioned in a report how BDA is implemented in large firms and makes their processes efficient. In another study, Liu et al. (2021) claimed that retailers can bring improvement in the overall experience of the customers by using big data. A few scholars (Johnson et al., 2017; Vaio et al., 2021) also analyzed the role of BDA in large organizations and found that it helps in identifying opportunities. In Singh and Kassar (2019) study, they revealed that BDA is proved to be helpful for the large firms in exploiting new opportunities through deploying existing resources.

Several prior studies have attempted to investigate the relationship between firm performance and BDA capabilities and highlighted the importance of decision-making based on the evidence. A study found a positive significant association between firm performance and IT capabilities reported the mediating effects of decision-making performance and business process performance on the relationship between firm performance and information management capabilities. In this study, they analyzed the role of BDA capabilities on firm performance using business strategy alignment as a mediator. Furthermore, another study empirically examined the data obtained from the firms in Italy. The study revealed the significance of big data in decision-making to achieve better performance. For the successful implementation of BDA capabilities in the healthcare sector, five strategies were suggested. These are: (i) creating a culture of information sharing, (ii) implementing big data governance, (iii) providing BDA-related training, (iv) developing new ideas for business through BDA, and (v) incorporating cloud computing in the BDA of the organization. In addition, a positive and significant association among big-data-driven actions, big-data-savvy teams, and firm performance was examined in a study. In their study, those techniques and skills were examined that are usually adopted by big data-savvy teams. Such teams consist of a group of professionals with diversified knowledge and skills. In this study, BDA capabilities (human skills, intangible, and tangible skills) and innovation were found to be positively associated with the dynamic capabilities as a mediator. The study also highlighted the role of environmental factors and their role as a moderator on the relationship between innovation and BDA capabilities. An organization in which resources are deployed collaboratively and decision-making is usually done based on evidence will likely result in performance gains for the firms. Recently, a different approach was adopted for analyzing BDA’s effects on firm performance. For this purpose, a framework was developed involving four domains explaining how adoption and implementation of BDA approaches may cause failure to the firm. Therefore, those firms which integrate simple data and develop ordinary capabilities have the tendency for business failure.

Based on the literature reviewed, the study has proposed the following hypothesis:

***H1: Organizational BDA capabilities have a significant impact on the performance of the firms.***
***H1a:** Big data contextualization capability has a significant impact on the performance of the firms.*
***H1b:** Big data democratization capability has a significant impact on the performance of the firms.*
***H1c**: Big data execution capability has a significant impact on the performance of the firms.*

The term organizational agility is defined as the business’s ability to renew itself and quickly respond (Ardito et al., 2019). Organizational agility is derived from the ability of an organization to adjust to the new situations with the existing set of resources/assets. In fact, organizational agility and its dynamic capabilities are often connected. An organization can significantly increase its agility when the information processing procedures and architectures do not become a burden to the organization’s culture of dynamism (Eslami et al., 2021). Organizational agility refers to the permanently available and learned dynamic capability which can be efficiently and quickly performed when needed in a certain way under an uncertain market environment for improving the business performance. Several researchers argued that organizational agility positively influences business performance. Several practitioners and information system scholars have pointed out that BDA capabilities can be integrated into the business operations (Gao, 2013; Wamba and Mishra, 2017). The BDA capable firms are considered as the information systems which collect huge data from the business processes, carry out data analysis, and then share the obtained results with the involved parties (Wamba and Mishra, 2017). Just like traditional BPM systems, these firms also aim to achieve innovation, effectiveness, and efficiency. However, these firms are different when it comes to fundamental aspects. According to Gao (2013), integrating BDA into the business processes provides situational awareness to the participants, besides, it also allows them in shaping their responses. Furthermore, in order to get advantage from big data, the BDA firms must be agile, automatic, analytical, and adaptive (Vossen, 2014; Vera-Baquero et al., 2015). It also improves the capability of the system to carry out advanced analysis besides performing multiple business data processing (Wamba and Mishra, 2017). These systems when integrated in multiple organizations promote information exchange within the organizations (Vera-Baquero et al., 2015). If the firms are adaptive in nature, they can develop complex data and can become more adaptable to the situations. In addition, such firms also promote agility, dynamism, and organizational flexibility. Such systems allow to successfully provide customer satisfaction, improve collaboration between organizations, and to capture responses to the market changes (Scuotto et al., 2017; Wamba and Mishra, 2017). Thus, the implementation of such systems depends on the market capitalization agility of the organization. Lu and Ramamurthy (2011) argue that the relationship between operational adjustment agility and such systems can also be observed. BDA capable firms have this unique ability to analyze the data on internal business processes and on the basis of this data, they allow firms to promote the efficiency of these processes. BDA firms also have the potential to improve exploitation capabilities as well as the organizations’ exploration capabilities (Sivarajah et al., 2017). The significant number of studies have been carried out concerning the significance of information systems and their importance in ambidextrous firms, however, not enough studies have been conducted in context to BDA capable firms. Such firms, significantly contribute to increase agility in ambidextrous organizations. Besides, they are also capable of influencing the organization’s operational adjustment agility and market capitalizing agility. Therefore, we propose:

***H2: Organizational BDA capabilities have a significant impact on the organizational agility.***
***H2a:** Big data contextualization capability has a significant impact on the organizational agility.*
***H2b:** Big data democratization Capability has a significant impact on the organizational agility.*
***H2c**: Big data execution capability has a significant impact on the organizational agility.*

This notion is associated with the idea that free-flowing information in an organization develops understanding among people about what they are required to do. These findings are relevant to the literature on the significance of BDA capabilities. Researchers argue that collecting information through BDA infrastructure facilitates the BDA skilled personnel and managers in quickly responding and making decisions, which ultimately affect the ability of an organization to respond (Şerban, 2017). All such findings suggest the impact of organizational BDA capabilities on the agility of a firm (Hyun et al., 2020). Moreover, organizational performance is often linked to organizational agility, which suggests that organizations having the ability of agility and adaptability can thrive and come out of difficult situations. Therefore, we propose:

***H3: Organizational agility has a significant impact on the performance of the firms.***

***H4: Organizational agility of the firm mediates the relationship between the organizational BDA capabilities and performance of the firms.***
*H4a: Organizational agility of the firm mediates the relationship between the organizational Big Data Contextualization capability and performance of the firms.*
*H4b: Organizational agility of the firm mediates the relationship between the organizational Big Data Democratization Capability and performance of the firms.*
*H4c: Organizational agility of the firm mediates the relationship between the Big Data Execution Capability and performance of the firms.*

Several distinguished scholars have suggested to view IT from a broader perspective to understand IS investments and its business value and assess the productive paradox with regards to IT (Bharadwaj, 2000; Santhanam and Hartono, 2003; Bhatt and Grover, 2005). According to these scholars, IT capability should be of prime focus, and Bharadwaj (2000) defined IT capability as “the ability of a firm to deploy and mobilize a combination of IT resources with other organizational capabilities and resources.” Most IT capability-based studies (Bharadwaj, 2000; Santhanam and Hartono, 2003) have integrated RBV that is derived from the firm’s strategic management. The extant literature indicates that organization can successfully gain a competitive advantage by employing valuable, inimitable, and distinctive capabilities and resources (Bhatt and Grover, 2005). Santhanam and Hartono (2003) argued that IT capability concept assumes that generally there is an easy replication of resources, but a firm’s distinctive capabilities cannot easily be replicated, thereby leading a firm toward achieving sustained competitive advantage. Another study (Aral and Weill, 2007) claimed that investments in IT assets are driven by the organizational strategies which add value to the firm’s performance in various dimensions, particularly in the context of strategic management. In this study, we used IT capability, i.e., IT functionality for supporting and shaping business strategy to successfully achieve strategic integration (van Der Zee and De Jong, 1999). In addition, Porter and Millar (1985) also argue that through casual ambiguity, social complexity, and path dependency, the original capability can ultimately result in a competitive advantage. Therefore, this study considers BDAC as a key capability that helps to achieve sustained competitive advantage in the context to big data (Davenport, 2006; Davenport et al., 2007; McAfee et al., 2012; Goes, 2014).

Several prior studies have proposed various types of IT capabilities, such as IT capability is defined by Bhatt and Grover (2005) in terms of heterogeneity, imperfect mobility, and value. In view of these scholars, heterogeneity and value of IT capability are essential, and imperfect mobility is an important condition to achieve competitive advantage. Furthermore, they also proposed three different capabilities for understanding IT-based sources of competitive advantage, these are: competitive capability (i.e., IT business expertise quality), dynamic capability (i.e., organization’s learning intensity), and value capability (i.e., IT infrastructure quality). Another study (Kim et al., 2012) conceptualized the IT capability of a firm using a social-materialistic approach. They observed that IT capability is driven by IT personnel capability, IT infrastructure capability, and IT management capability. Developing models based on social-materialism stresses upon the interconnection between these capabilities (IT personnel capability, IT infrastructure capability, IT management capability), which is in contradiction to the traditional IS approaches, that characterize IT capability as unrelated and a unidirectional concept.

Authors found that IT capability and firm performance are positively related. This finding is in line with several prior studies which analyzed how IT capability is related to firm agility, firm performance, stock market returns, etc. (Lin, 2007). Similarly, we also reviewed BDA capabilities and found three dimensions, i.e., personnel, infrastructure, and management capabilities. In McAfee et al.’s (2012) study, they suggested that some of the critical capabilities in the data economy are technology infrastructure, corporate decision-making, and personnel management. Another study (Kiron et al., 2014) reported analytics platform, analytics skills of employees, and organization culture as the key BDA dimensions. Moreover, in Davenport et al.’s (2012) study, they pointed out an interrelationship between people, technology, and management under a big data environment and suggested that it facilitates in achieving firm performance. Barton and Court (2012) also supported these dimensions and the relationship among them. They highlighted the essential role of technology capability for data exploring and management; the role of management capability for improving decision models, and the importance of data science capability for understanding, creating, and implementing analytics models.

Several distinguished scholars highlighted the significance of viewing IT through a broader perspective for it allows to determine the IS investment’s business value and ways to handle IT productive paradox. In study, he emphasized the significance of IT capability and referred IT capability as the ability of a firm to utilize and mobilize IT resources combining with other capabilities and resources. Resource-based view (RBV) is a commonly used perspective when it comes to IT capability and is derived from strategic management. Research in this area suggests that an organization can use inimitable, distinctive, and valuable capabilities and resources to achieve a competitive advantage. They argued that a firm’s distinctive capabilities cannot be replicated and, thus, lead the firm to the competitive advantage. Basically, this assumption governs the whole concept of IT capability.

Scholars in the area of strategic management suggested that firms’ strategies determine the investments that are to be made in various IT assets. Besides, these strategies also provide value in various dimensions. For achieving strategic integration in this research, IT capability will be integrated for IT functionality, i.e., for supporting and shaping the business strategy. They argued that through causal ambiguity, social complexity, and path dependency, the original capabilities are likely to result in sustained competitive advantage. Prior studies also confirmed and suggest BDAC as important because it brings about the competitive advantage to the firm in the organizational setting with big data.

Scholars have proposed various typologies concerning IT capabilities, such as in study, IT capability is categorized as imperfect mobility, heterogeneity, and value. They further argued that in order to achieve a competitive advantage, IT capability heterogeneity and value are essential; however, for the sustained advantage, imperfect mobility is essential. Also, conceptualized capabilities as competitive capability, dynamic capability, and value capability are also essential. Furthermore, in this study, they considered a sociomaterialistic view and suggested that IT capability is a function of personnel capability, IT infrastructure capability, and IT management capability. Unlike traditional IS approaches, which conceptualize IT capability as unrelated or unidirectional, the sociomaterialism-based modeling emphasizes the integration of the discussed IT capabilities. Furthermore, a positive relationship was also reported among the firm’s financial and business process performance with the IT capability. Similar findings were reported in the literature, where IT capability’s relationship has been observed with stock market returns, firm performance, and firm agility. Therefore, a BDA capabilities literature was also reviewed which presented dimensions like the infrastructure, personal capabilities, and management capabilities dimensions. Such as, according to, the three critical capabilities are corporate decision making, technology infrastructure, personnel management in context to the data economy. Furthermore, the analytical skills of employees and analytics platforms are identified as the core BDA dimensions. In study, they pointed out the three interlinked dimensions namely: people, technology, and management. These dimensions under big data environment provide support in improving the firm performance. Findings were also consistent with these BDA dimensions. They also highlighted the significance of management capability in optimizing decision models. Besides, the capability of data science helps in developing, understanding, and in the application of analytics models, while technology capability helps in managing and exploring various types of data. Based on the literature reviewed the study has proposed the following hypothesis:

***H5: IT capability of the firm mediates the relationship between the organizational BDA capabilities and performance of the firms.***
*H5a: IT capability of the firm mediates the relationship between the organizational big data contextualization capability and performance of the firms.*
*H5b: IT capability of the firm mediates the relationship between the organizational big data democratization capability and performance of the firms.*
*H5c: IT capability of the firm mediates the relationship between the big data execution capability and performance of the firms.*

## Research Methodology

This study has been carried out in China to investigate the effect of BDMC on competitive advantages performance (CAP) of SMEs who are related to manufacturing business. This study adopted the quantitative method of research with a cross-sectional technique. This study employed a questionnaire to gather the data as a research instrument. This study has used the purposive sampling method by keeping in mind the context of study. Employees of the Chinese SMEs that were at least 10 years old were the population of this study. The researchers select the sample size by using the recommendations of Kyriazos (2018). According to the suggestion of Kyriazos (2018), the sample size of the respondent should be 300 because it is assumed a good sample; however, the 50 respondents are supposed a weaker sample, 100 respondents are reflected a weak sample, while a sample of 200 respondents considers an adequate. Thus, this study chooses a 300 respondents as a sample. A total of 500 survey questionnaires were disseminated among the selected population. Out of 500 distributed questionnaires, only 312 questionnaires were returned and usable for the purpose of analysis. It shows that the response rate was 62.4%. According to Sabir et al. (2019), the average response rate in the management sciences study is 56% and this study achieved the adequate level of response rate.

## Measurement

A 18-item scale is adopted in this study, which has also been previously adopted by Wamba et al. (2017) to measure the BDA capabilities of an organization. Following prior research by Mikalef and Pateli (2017), organizational BDA capabilities are taken as a second-order construct. The variables that were selected for this study were derived from first-order constructs, namely, BDA management capabilities (with seven items), BDA personnel expertise (with five items), and BDA infrastructure (with six items). In this study, the BDA infrastructure-related statements were used as latent constructs that are based on compatibility (i.e., software applications can be easily integrated at various analytical platforms), connectivity (i.e., our industry owns required analytical systems in comparison to our industry rivals), and modularity (i.e., in developing new system, reusable software modules are used widely in this context). Besides, statements that were used as latent constructs of BDA management capabilities include statements that are related to coordination (e.g., line people and business analysts in our organization meet on regular basis for the purpose of discussing key issues), control (i.e., analytics development responsibility is clear in our organization), decision-making (i.e., while making decision-related to business analytics investment, we also measure its impact on the employees’ work productivity), and planning (e.g., innovative opportunities are continuously examined to be adopted in business analytics). Last, the BDA personnel expertise-related statements that were used as variables include those which are linked with business knowledge (e.g., analytics personnel in our organization are capable enough to identify business problems, and find appropriate solutions), relational knowledge (i.e., close coordination is practiced by the analytics personnel to develop client relationships), and technical knowledge (i.e., analytics personnel in our organization are capable enough to regulate decision support system, such as, data warehousing, data mining, expert systems, and artificial intelligence, etc.) (Wamba et al., 2017).

Furthermore, Navarro et al. (2016) scale with six-items was also used in this study for measuring organizational agility. This scale has also been previously used by researchers to analyze how technologies and information systems affect organizational agility. In addition, we adopted a 12-item scale [e.g., organization performs well to satisfy customers by Gomes and Facin (2019) for organizational performance measurement].

## Analysis and Results

The research model was being analyzed by employed “partial least squares” technique through statistical software the Smart PLS software version 3 (Ringle et al., 2015). The two-level analyses method suggested by Henseler et al. (2014) and Ramayah et al. (2017) was used for the analysis of data. The measurement model was assessed in the first stage and then the structural model was estimated by this study (Hair et al., 2019; Shehzadi et al., 2020).

### Measurement Model Evaluation

**FIGURE 1:**
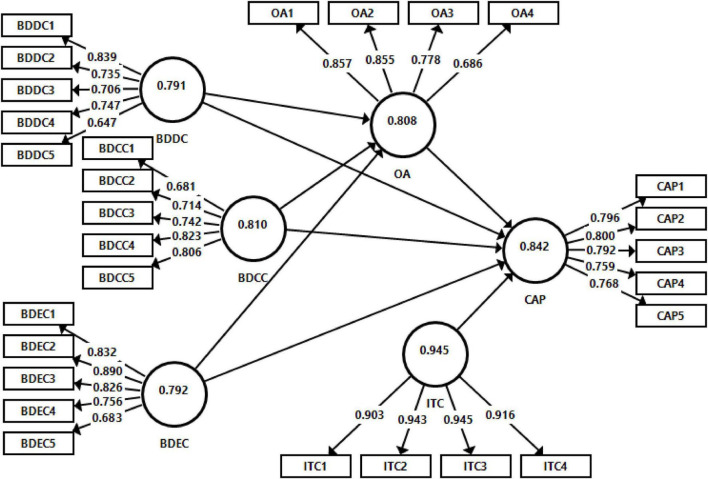
Measurement model assessment.

The measurement model was estimated to assess the validity of constructs. Estimation of measurement model used to examine the discriminant and convergent validity. The values of outer loadings, “average variance extracted” (AVE) and “composite reliability” (CR), are applied to examine the convergent validity of variables (Henseler et al., 2014; Nisar et al., 2021). However, the loadings must be greater than 0.5 and the value of CR and AVE must be above from 0.7 to 0.5, respectively, to validate the convergence of the model (Sarstedt et al., 2020). To validate the model from the discriminant point, two measures are used, one is HTMT ratio and the other one is the Shiau et al. (2019) criteria. HTMT ratio must be higher than 0.85 to established discriminant validity. The results of the measurement model are given in Figure 1 and Tables 1–3.

**TABLE 1:** Internal consistency, convergent validity, composite reliability, and average variance extracted (AVE).

Construct	Indicators	Loadings	Cronbach’s alpha	Composite reliability	AVE
Big Data Contextualization Capability (BDCC)	BDCC1	0.681	0.810	0.868	0.570
	BDCC2	0.714			
	BDCC3	0.742			
	BDCC4	0.823			
	BDCC5	0.806			
Big Data Democratization Capability (BDDC)	BDDC1	0.839	0.791	0.856	0.544
	BDDC2	0.735			
	BDDC3	0.706			
	BDDC4	0.747			
	BDDC5	0.647			
Big Data Execution Capability (BDEC)	BDEC1	0.832	0.792	0.854	0.563
	BDEC2	0.890			
	BDEC3	0.826			
	BDEC4	0.756			
	BDEC5	0.683			
Competitive Advantages Performance (CAP)	CAP1	0.796	0.842	0.888	0.613
	CAP2	0.800			
	CAP3	0.792			
	CAP4	0.759			
	CAP5	0.768			
Information Technology Capability (ITC)	ITC1	0.903	0.945	0.961	0.859
	ITC2	0.943			
	ITC3	0.945			
	ITC4	0.916			
	ITC1	0.903			
Organizational Agility (OA)	OA1	0.857	0.808	0.874	0.635
	OA2	0.855			
	OA3	0.778			
	OA4	0.686			

**TABLE 2:** Fornell–Larcker criterion.

	BDCC	BDDC	BDEC	CAP	ITC	OA
BDCC	0.755					
BDDC	0.673	0.738				
BDEC	0.386	0.508	0.750			
CAP	0.638	0.710	0.505	0.783		
ITC	0.341	0.562	0.502	0.468	0.927	
OA	0.425	0.568	0.515	0.522	0.650	0.797

**TABLE 3:** Heterotrait-monotrait (HTMT) ratio.

	BDCC	BDDC	BDEC	CAP	ITC	OA
BDCC						
BDDC	0.819					
BDEC	0.505	0.610				
CAP	0.766	0.843	0.578			
ITC	0.392	0.634	0.558	0.523		
OA	0.517	0.696	0.607	0.624	0.734	

Table 1 presents that this study meets the criteria of convergent validity according to the suggestion of Sarstedt et al. (2019). The value of loadings is above 0.6, the CR values are greater than 0.7, and the AVE values are higher than 0.5.

Table 2 illustrates that this model achieves the discriminant validity according to the recommendations of Shiau et al. (2019). The values of the AVE square root of all the variables are higher than the correlations with other variables.

Table 3 indicates that HTMT ratios are lesser than 0.85; therefore, this model achieves the discriminant validity according to HTMT criteria.

### Structural Model Evaluation

**FIGURE 2:**
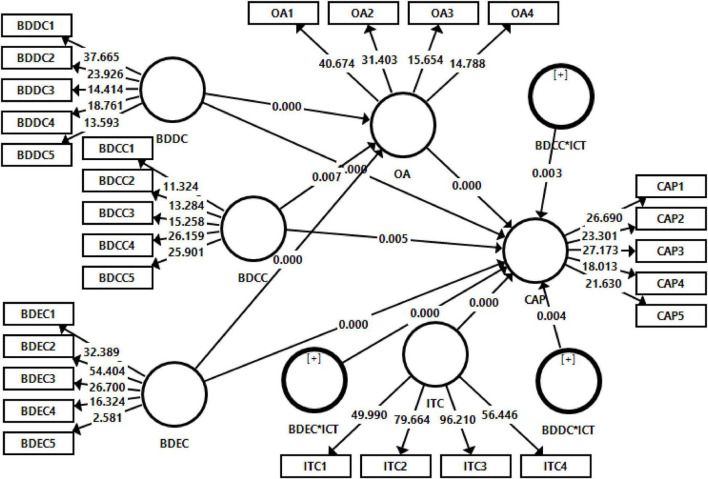
Structural model assessment.

The bootstrapping procedure was adopted to estimate the hypotheses, to check the effect of independent variables on the dependent variable, and to check the mediation and moderation effects (Sarstedt et al., 2019). A resamples of 1,000 in bootstrapping were employed to estimate the hypotheses. The results of the structural model are given by Figure 2 and Tables 4–6.

**TABLE 4:** Structural model assessment (direct effect results and decision).

Hypotheses	Relationship	Beta	STD	*T* value	*P*-values
H_1a_	BDCC→CAP	0.195	0.068	2.849	0.005
H_1a_	BDDC→CAP	0.442	0.082	5.388	0.000
H_1a_	BDEC→CAP	0.114	0.052	2.192	0.000
H_2a_	BDCC→OA	0.201	0.021	2.741	0.007
H_2b_	BDDC→OA	0.381	0.057	3.323	0.008
H_2c_	BDEC→OA	0.227	0.063	4.121	0.000
H_3_	OA→CAP	0.401	0.037	2.901	0.000

**TABLE 5:** Structural model assessment indirect effect (mediation effects).

Hypotheses	Relationship	Beta	STD	*T* value	*P*-values
H_4a_	BDCC→OA→CAP	0.232	0.086	2.698	0.001
H_4b_	BDEC→OA→CAP	0.111	0.022	5.045	0.006
H_4c_	BDDC→OA→CAP	0.214	0.027	7.926	0.000

**TABLE 6:** Structural model assessment (moderation effects).

Hypotheses	Relationship	Beta	STD	*T* value	*P*-values
H_5a_	BDCC*ICT→CAP	0.378	0.073	5.168	0.003
H_5b_	BDDC*ICT→CAP	0.212	0.073	2.904	0.004
H_5c_	BDEC*ICT→CAP	0.169	0.077	2.191	0.000

Table 4 indicates the direct effects hypotheses estimation results. Findings indicated that big data contextualization capability has a positive and significant effect on CAP (β = 0.195, *t* = 2.849) and big data democratization capability also positively and significantly effective to CAP of Chinese manufacturing firms (β = 0.442, *t* = 5.388). Furthermore, big data execution capability also has a significant influence on CAP (β = 0.114, *t* = 2.192). Thus, H1, H2, and H3 are supported.

The results of mediating analysis are shown in Table 5. Results elucidated that organizational agility has a significant mediation effect in the association of Contextualization Capability and CAP (β = 0.232, *t* = 2.698). Organizational agility also significantly mediated the association of Big Data Democratization Capability with CAP (β = 0.111, *t* = 5.045). Moreover, the association between Big Data Execution Capability and CAP significantly mediates with organizational agility (β = 0.214, *t* = 7.926).

Table 6 indicates the output of moderation analysis. Results indicated that Information Technology Capability significantly moderates the association of contextualization capability with CAP (β = 0.378, *t* = 5.168). Information technology capability also has a significant moderating role on the association of big data democratization capability with CAP (β = 0.212, *t* = 2.904) and it also has a moderating role on the relationship of big data execution capability with CAP (β = 0.169, *t* = 2.191).

## Discussion and Conclusion

Findings obtained in this study show that a large organization’s structure can be reshaped with organizational BDA capabilities. It is found that meaningful information is extracted from the big dataset using required skills, processes, and infrastructures, which enable large organizations to pursue better opportunities available in the market. This study also provides useful insights for the organizations on the firm performance and big data relationship. This study significantly contributed to the BDA capabilities literature. In this study, the role of agility is analyzed for collecting data from the big dataset, and how organization performance improves through agility (Korherr and Kanbach, 2021). The outcomes and findings obtained from this research also add to the dynamic capabilities and big data literature since a moderated multimediation model is proposed in this study, which develops understanding of the interrelationships and complex dynamics in this context. The findings of this study are consistent with Altay et al. (2018) study indicating that organizational ability to strive under competitive situations and organizational dynamic capabilities are related.

It is noteworthy that BDA infrastructure promotes organizational agility, especially in large firms. However, this finding contradicts with the notion of IMS. Prior studies such as Acosta et al. (2018) have often emphasized the rigidness of IMS (BDA infrastructure) and it hinders organizational dynamism. Such discrepancy may occur because the operational performance of BDA infrastructure is better in comparison to the traditional IMS because of the technical features of BDA infrastructure. Regardless of the type of infrastructure, such as data lakes, Internet of Things, or cloud computing, BDA infrastructures operate on the leaner architectures as compared to the traditional systems. It can be due to the fact that BDA infrastructure improves the large organizations’ ability to identify, and exploit opportunities by providing enough information and then enables them to quickly respond to the changes. Therefore, due to the abovementioned reasons, large organizations are regarded as less agile and more rigid as compared to the SMEs, and BDA infrastructures can address this problem well as compared to the traditional IMS (Mauro et al., 2018). In fact, communication among organizational units also improves by integrating BDA infrastructures, as it helps them in timely responding to the issues and opportunities (Vaio et al., 2021).

Several business value-based studies also found mixed findings, such as “IT productive paradox.” A few researchers (Roach et al., 1987; Solow, 1987; Strassmann, 1990) argued that the idea “IS investments result in the improvement in efficiency and effectiveness of operations” cannot always be true. On the contrary, a few scholars (Barua et al., 1995, 2004; Brynjolfsson and Yang, 1996) also found firm performance and IS investments as positively related in their studies. Such findings are indicative of the fact that failure to obtain positive linkage among firm performance and IS investment may occur due to several other factors such as time lags between the generated business value from such investments and IS investments, lack of appropriate data, benefit analysis of IS investments, and no assessment for ITs indirect benefits (Brynjolfsson and Yang, 1996; Brynjolfsson and Hitt, 2000; Devaraj and Kohli, 2003; Anand et al., 2013). Indeed, in context to this research, scholars (Mooney et al., 1996; Anand et al., 2013) claimed that several intermediate variables can be responsible to mediate the effects of IT on the organizational performance. They also suggested to consider the impact of IT resources using a broader perspective, i.e., considering various dimensions while studying the business value of IT capabilities or IT. Therefore, this study aims to extend the literature by analyzing those factors which lead to better organizational performance, resulting from BDA investments.

Big data analytics through its strategic and operational potential has entirely changed the game and thus improves the effectiveness and efficiency of the businesses. Recent research (e.g., Germann et al., 2014) on BDA has reported a positive association between firm performance and the use of customer analytics. Such as Brands (2014) stated that BDA enables the use of data lens for managing and analyzing firm strategy. Hagel (2015) also acknowledged and highlighted the significance of BDA in the firm’s decision-making processes. According to Liu et al. (2014), BDA differentiates high-performing firms from the low-performing firms, since it increases organizational revenue, minimizes its customer acquisition by 8 and 47%, respectively, and enables firms to develop capabilities, such as forward-looking and proactiveness to gain such benefits (Liu et al., 2014). Literature review suggests various examples where BDA has been used by firms, one such example is the case of Target Corporation. The company used BDA for tracking the purchasing behavior of their customers through using a loyalty card program which thus enabled them to predict the future buying trend of their customers. Amazon.com also took advantage from BDA, in fact, Amazon.com generated 35% of its purchases based on BDA, i.e., by suggesting its customers with personalized purchase recommendations (Wills, 2014).

## Managerial Implications

This study provides senior management with the insights about how firm performance can be improved through BDA capabilities. It also points out areas that lead to the maximum utilization of BDA capabilities. Pakistan likes other emerging markets needed to create business environment characterized with technological advancements. Thus, this study emphasizes and points out the BDA capabilities of firms in Pakistan and how they are related to the firm performance. This will allow the top management to determine the weaknesses and strengths of the firm and devise strategies accordingly. The operational efficiency of sample firms can be improved by integrating IT and IS systems through non-financial and financial investment for developing the BDA capabilities.

## Data Availability Statement

The raw data supporting the conclusions of this article will be made available by the authors, without undue reservation.

## Author Contributions

Both authors listed have made a substantial, direct, and intellectual contribution to the work, and approved it for publication.

## Conflict of Interest

The authors declare that the research was conducted in the absence of any commercial or financial relationships that could be construed as a potential conflict of interest.

## Publisher’s Note

All claims expressed in this article are solely those of the authors and do not necessarily represent those of their affiliated organizations, or those of the publisher, the editors and the reviewers. Any product that may be evaluated in this article, or claim that may be made by its manufacturer, is not guaranteed or endorsed by the publisher.
